# Depressive symptoms and sleep problems as risk factors for heart disease: a prospective community study

**DOI:** 10.1017/S2045796019000441

**Published:** 2019-08-20

**Authors:** S. S. Deschênes, R. J. Burns, E. Graham, N. Schmitz

**Affiliations:** 1 Department of Psychiatry, McGill University, Montreal, Canada; 2 Douglas Mental Health University Institute, Montreal, Canada; 3 Department of Psychology, Carleton University, Ottawa, Canada; 4 Department of Epidemiology and Biostatistics, McGill University, Montreal, Canada

**Keywords:** Depression, epidemiology, health outcomes, sleep

## Abstract

**Aims:**

The goals of the present study were to examine the associations between depressive symptoms, sleep problems and the risk of developing heart disease in a Canadian community sample.

**Methods:**

Baseline data were from the CARTaGENE study, a community health survey of adults aged 40–69 years in Quebec, Canada. Incidence of heart disease was examined in *N* = 33 455 participants by linking survey data with administrative health insurance data. Incident heart disease was identified using the World Health Organization's International Classification of Diseases, 9th or 10th edition (ICD-9 and ICD-10) diagnostic codes for heart disease. Sleep problems were assessed with diagnostic codes for sleep disorders within the 2 years preceding the baseline assessment. Average sleep duration was assessed by self-report. Depressive symptoms were assessed with the nine-item Patient Health Questionnaire.

**Results:**

In total, 2448 (7.3%) participants developed heart disease over an average follow-up period of 4.6 years. Compared to those without depressive symptoms and with no sleep disorders, those with elevated depressive symptoms and a sleep disorder (HR = 2.60, 95% CI 1.83–3.69), those with depressive symptoms alone (HR = 1.40, 95% CI 1.25–1.57) and those with sleep disorders alone (HR = 1.33, 95% CI 1.03–1.73) were more likely to develop heart disease. Test of additive interaction suggested a synergistic interaction between depressive symptoms and sleep disorders (synergy index = 2.17 [95% CI 1.01–4.64]). When sleep duration was considered, those with long sleep duration and elevated depressive symptoms were more likely to develop heart disease than those with long sleep alone (HR = 1.77, 95% CI 1.37–2.28; and HR = 1.16, 95% CI 0.99–1.36, respectively).

**Conclusions:**

Depression and diagnosed sleep disorders or long sleep duration are independent risk factors for heart disease and are associated with a stronger risk of heart disease when occurring together.

## Introduction

Heart disease is a leading cause of death worldwide (Lozano *et al*., 2012; Wang *et al*., 2016). Although several biological and behavioural risk factors for the development of heart disease have been identified, including low HDL cholesterol (Sniderman *et al*., 2011), hypertension (Ettehad *et al*., 2016), diabetes (Huxley *et al*., 2006; Sarwar *et al*., 2010), obesity (McGee, 2005; Bogers *et al*., 2007) and physical inactivity (Sattelmair *et al*., 2011), a better understanding of modifiable risk factors, and their interactions, could be beneficial for the prevention of heart disease. Sleep may be an additional modifiable risk factor for heart disease. Sleep disorders, including sleep-disordered breathing, insomnia and hypersomnia, have been linked with the incidence of heart disease (Katz and McHorney, 1998; Bradley and Floras, 2009; Dikeos and Georgantopoulos, 2011; Sofi *et al*., 2014; Javaheri and Redline, 2017), as have short and long sleep durations (Cappuccio *et al*., 2011; Itani *et al*., 2017; Jike *et al*., 2018). For instance, insomnia symptoms were associated with a 45% increased risk of incident or death from cardiovascular disease in a meta-analysis (Sofi *et al*., 2014).

Increasing evidence suggests that depression is another potentially modifiable risk factor for heart disease (Scott, 2014). For example, one study found that approximately 30% of the risk of developing myocardial infarction was reduced when psychological factors such as depression were considered (Yusuf *et al*., 2004). The association between depression and the development of cardiovascular disease was also demonstrated in the meta-analyses of studies from general populations (Nicholson *et al*., 2006; Van der Kooy *et al*., 2007; Gan *et al*., 2014; Wu and Kling, 2016; Wium-Andersen *et al*., 2019). Depressed mood moderately increased the risk for cardiovascular diseases; notably, the magnitude of the risk was equivalent to that for smoking and diabetes (Van der Kooy *et al*., 2007). Studies have also demonstrated that subsyndromal depression is associated with an increased likelihood of developing heart disease (Jiang *et al*., 2002).

Taken together, sleep problems and depression have been identified as risk factors for heart disease. However, there is an overlap between depressive symptoms and sleep problems. Depression is often accompanied by impairments in sleep quality or short or long sleep duration (Alvaro *et al*., 2013), and short or long sleep is a diagnostic criterion for major depressive disorder (American Psychiatric Association, 2013). It is possible that depression and sleep problems exacerbate a common pathway, which might result in a substantially elevated risk of heart diseases in people with both conditions. Prior research has linked depression subtypes with the risk of health conditions such as diabetes (Schmitz *et al*., 2016) and it is possible that depression with sleep problems is another subtype of depression strongly linked with heart disease. Despite the close associations between sleep and depression, it is unclear if these factors interact with each other to increase the risk of heart disease (Laugsand *et al*., 2011; Redline and Foody, 2011). Prior studies have focused on depression and sleep problems as independent predictors of heart disease. No prospective study, to our knowledge, has evaluated the interactions between depression and sleep problems as potential risk factors for the development of heart disease.

The goals of the present study were to examine the associations between depressive symptoms, diagnosed sleep disorders or short and long sleep durations, and the risk of developing heart disease during a 7-year follow-up period in a Canadian community sample. We hypothesised that the strongest associations with heart disease would be found among those with both elevated depressive symptoms and a diagnosed sleep disorder compared to those with no/low depressive symptoms and no diagnosed sleep disorder. We also hypothesised that the strongest association with heart disease would be seen in those with both elevated depressive symptoms and either short or long sleep durations.

## Method

### Sample and procedures

Baseline data were derived from the CARTaGENE cohort study (https://cartagene.qc.ca/), a population-based health survey of residents in Quebec, Canada, that was conducted in 2009–2010 (phase A, *n* =  20 004) or in 2012–2015 (phase B, *n* = 23 000). Participants were between 40 and 69 years of age at baseline and were recruited from the public health insurance database in Quebec, the *Régie de l'Assurance Maladie du Québec* (RAMQ) (Awadalla *et al*., 2013). Follow-up data about heart disease were collected by linking CARTaGENE participants with diagnostic codes from the RAMQ billing database. This study was approved by the Douglas Mental Health University Institute Research Ethics Board and the St Justine Hospital Research Ethics. All participants provided informed consent to participate in the CARTaGENE survey and to have their data linked with the RAMQ database.

For the present study, CARTaGENE participants with self-reported cardiovascular disease at baseline were excluded based on a positive response to any of the following three questions: ‘*Has a doctor ever told you that you have had a myocardial infarction or a heart attack? Has a doctor ever told you that you had angina? Has a doctor ever told you that you have had a stroke?*’ Participants also needed to have complete data on the depression assessment. In total, there were 33 925 CARTaGENE participants without self-reported heart disease at baseline and with complete depression data. In addition, only those that were able to be linked with the RAMQ database (*n* = 459 excluded) were included in the present study sample. The final sample for the present study was *n* = 33 455 participants.

### Measures

Depressive symptoms were assessed with the Patient Health Questionnaire (PHQ-9) (Kroenke *et al*., 2001), a nine-item questionnaire about the frequency of depressive symptoms experienced in the past 2 weeks. The PHQ-9 showed good internal consistency in our sample (*α* = 0.82). Responses were summed and a score of 6 or higher was classified as elevated depressive symptoms (Lamers *et al*., 2008). Previous studies have shown good performance using this cut-off score (Zuithoff *et al*., 2010; Schmitz *et al*., 2016). A sensitivity analysis was conducted using a cut-off score of 10 or more for moderate-to-severe depressive symptoms (Kroenke *et al*., 2001).

Sleep disorders in the 2 years prior to the CARTaGENE survey were identified using diagnostic codes from the RAMQ billing database. Diagnostic codes were based on the World Health Organization's International Classification of Diseases, 9th or 10th edition (ICD-9 and ICD-10, respectively) and included those for sleep apnoea and sleep-related disorders (insomnia, hypersomnia, parasomnia; ICD-9 codes 307.4, 327.0, 327.2, 327.1, 327.3, 327.4, 333.94, 292.8, 291.8, 780.5 and 347.0; ICD-10 codes G25.81, G47.0, G47.1, G47.30, G47.31, G47.8, F51.0, F51.1, F51.8 and F51.9). At the time of data analysis, the RAMQ database applied ICD-9 codes for outpatient medical service visits, including visits with general practitioners, and ICD-10 codes for hospital visits.

Self-reported sleep duration was assessed with the question ‘On average how many hours per day do you usually sleep including naps?’ Short sleep duration was defined as 6 h or less, normal sleep duration was defined as more than 6 h and less than 9 h, and long sleep duration was defined as 9 h or more. These categories were based on the consensus recommendations of the American Academy of Sleep Medicine (Watson *et al*., 2015). Although differences between subjective and objective measures of sleep duration have been reported (Van Den Berg *et al*., 2008), self-reported sleep duration has demonstrated good concordance with objective measures of sleep duration using actigraphy or polysomnography (Signal *et al*., 2005).

Incidence of heart disease was assessed using diagnostic codes for coronary heart disease or heart failure in the RAMQ billing database (ICD-9 codes 410 [acute myocardial infarction], 411 [other acute and subacute forms of ischemic heart disease], 413 [angina pectoris], 414 [other acute and subacute forms of ischemic heart disease], and 428 [heart failure] or ICD-10 codes I21 [acute myocardial infarction] and I23 [haemopericardium as current complication following acute myocardial infarction]). The date of the first diagnosis or hospital admission for heart disease was recorded.

Covariates were assessed during the CARTaGENE baseline interview. Sociodemographic characteristics included age, sex, ethnicity and highest level of education achieved. Behavioural factors included smoking, frequency of alcohol consumption and physical activity (number of days per week spent participating in moderate and vigorous physical activity). Diabetes status and high blood pressure were assessed by self-report of receiving a diagnosis from a health professional. Low cholesterol levels were defined as having low high-density lipoprotein cholesterol (<1.03 mmol/l in men and <1.30 mmol/l in women) or self-reported cholesterol problems (‘*Has a doctor ever told you that your blood cholesterol was high?*’) for those with missing blood samples. Covariate selection was based on the variables included in the Framingham risk score (D’Agostino *et al*., 2008) as well as other risk factors for heart disease (Corrao *et al*., 2000; Varghese *et al*., 2016).

The number of missing values for the covariates was generally low (<6%), with one exception: information on physical activity was only available for 82% of the sample. We therefore imputed missing values for physical activity using the PROC MI procedure in SAS.

### Statistical analysis

Analyses were performed using SAS version 9.4. The incidence of heart disease during the follow-up period was estimated for four groups based on the presence/absence of diagnosed sleep disorders and depressive symptoms category. The groups were: (1) no sleep disorder and no/low depressive symptoms (NSND), (2) no sleep disorder and elevated depressive symptoms (NSD), (3) sleep disorder and no/low depressive symptoms (SND) and (4) sleep disorder and elevated depressive symptoms (SD). Cox proportional hazards regression models were used to examine the associations between the groups in unadjusted analyses and analyses adjusted for the covariates age, sex, education, ethnicity, smoking, alcohol consumption, physical activity, diabetes, high blood pressure and cholesterol. Hazard ratios with 95% confidence intervals are reported. Person-years were calculated from the date of the CARTaGENE baseline interview to the first date of a diagnosis of heart disease, death (any cause) or the end of the follow-up period (31 December 2016). The assumption of proportional hazards was examined graphically by comparing the observed Kaplan–Meier survival curves with the predicted Cox survival curves and by a goodness-of-fit statistical test (Kleinbaum and Klein, 2010).

The synergy index (*S*) was used to estimate the departure from additive risk for the interaction between depressive symptoms and sleep disorders on the risk of heart disease (VanderWeele and Knol, 2014). *S* was calculated as [HR for depressive symptoms and a sleep disorder−1]/([HR for depressive symptoms only−1] + [HR for sleep disorder only−1]), with an *S* > 1.00 indicating a greater than additive interaction effect (i.e., synergism) (Andersson *et al*., 2005).

In a second step, we estimated the risk of heart disease based on sleep duration and depressive symptoms with six groups: (1) normal sleep duration/no depressive symptoms, (2) short sleep duration/no depressive symptoms, (3) long sleep duration/no depressive symptoms, (4) normal sleep duration/depressive symptoms, (5) short sleep duration/depressive symptoms and (6) long sleep duration/depressive symptoms. A linear contrast was constructed to test whether the association between sleep duration and incidence of heart disease differed by depression status (comparing group 2 *v*. group 5; and group 3 *v*. group 6).

Several sensitivity analyses were conducted to estimate the robustness of the study findings. First, we examined the associations between diagnosed sleep disorders in the past 5 years prior to the baseline assessment. Second, we conducted an analysis that excluded the sleep item from the PHQ-9 questionnaire (‘*trouble falling asleep, staying asleep or sleeping too much*’), using a cut-off score of 6 or more for elevated depressive symptoms. Third, a more stringent PHQ-9 cut-off score of 10 or more was used. Fourth, survival bias was examined with a competing-risk regression by including all-cause mortality in the model as a competing event. For this analysis, we modelled the cumulative incidence of heart disease in the presence of the competing event (i.e., death) with the sleep and depressive symptom categories as predictors.

## Results

A total of 33 455 individuals were followed for a total of 155 732 person-years. There were 2448 (7.32%) incident cases of heart disease identified over a mean follow-up period of 4.6 (SD  =  1.70) years.

Table 1 describes the participant characteristics. The incidence rates were 14.9, 21.1, 18.6 and 30.2 per 1000 persons per years for the NSND, NSD, SND and SD groups, respectively. Most of those with a sleep disorder (90%) had a diagnostic code for unspecified sleep disturbance, whereas 12% of those with a sleep disorder had a diagnostic code for sleep apnoea. Demographic and clinical characteristics of those who did and did not develop heart disease are presented in Table 2. There were substantial differences in behavioural factors, clinical characteristics, depressive symptoms and sleep characteristics between those who developed heart disease and those who did not. Individuals who developed heart disease had a higher level of depressive symptoms, were more often diagnosed with sleep disorders and were more often long or short sleepers than those who did not develop heart disease.

**Table 1.** Sample characteristics stratified by depressive symptoms and sleep disorder groups

	Full sample *N* = 33 455	No/low depressive symptoms and no sleep disorder (*n* = 27 361)	No/low depressive symptoms and sleep disorder (*n* = 757)	Elevated depressive symptoms and no sleep disorder (*n* = 5060)	Elevated depressive symptoms and sleep disorder (*n* = 277)
Age (Mean, SD)	53.0 (7.9)	53.2 (7.9)	54.1 (7.7)	51.9 (7.4)	52.2 (7.7)
Sex					
Female, % (*n*)	57.1 (19 101)	56.0 (15 325)	52.6 (298)	63.8 (3227)	54.5 (151)
Education					
Less than high school					
High school, % (*n*)	20.5 (6832)	19.4 (5290)	18.8 (142)	26.2 (1318)	29.8 (82)
College/graduate studies/university, % (*n*)	79.5 (26 488)	80.6 (21 967)	81.2 (612)	73.8 (3716)	70.2 (193)
Ethnicity					
White, % (*n*)	88.7 (29 416)	89.4 (24 264)	91.7 (686)	84.5 (4221)	89.1 (245)
Smoking					
Current/occasional smoker, % (*n*)	16.7 (5571)	15.3 (4172)	13.1 (99)	24.6 (1240)	21.9 (60)
Former smoker, % (*n*)	39.3 (13 112)	39.7 (10 819)	48.9 (370)	36.0 (1813)	40.2 (110)
Never smoker, % (*n*)	44.0 (14 667)	45.0 (12 287)	38.0 (288)	39.4 (1988)	38.0 (104)
Alcohol					
Consumes alcohol daily, % (*n*)	11.3 (3769)	11.6 (3166)	11.9 (90)	9.7 (487)	9.5 (26)
Physical activity					
No moderate/vigorous activity, % (*n*)	19.2 (5481)	17.6 (4109)	21.7 (143)	26.9 (1156)	29.9 (73)
Diagnosed high blood pressure, % (*n*)	15.8 (5120)	15.2 (4043)	20.8 (153)	18.1 (874)	19.0 (50)
Treated high blood pressure, % (*n*)	12.7 (4069)	12.3 (3235)	17.3 (125)	14.0 (669)	15.4 (40)
Low HDL cholesterol or self-reported cholesterol problems, % (*n*)	35.1 (11 008)	34.0 (8715)	37.5 (275)	40.0 (1890)	49.6 (128)
Diagnosed diabetes, % (*n*)	7.0 (2350)	6.4 (1732)	9.8 (74)	10.0 (504)	14.6 (40)
Developed heart disease, % (*n*)	7.3 (2448)	7.0 (1908)	9.4 (71)	8.5 (432)	13.4 (37)
PHQ-9 scores (mean, SD)	2.8 (3.5)	1.6 (1.6)	2.1 (1.7)	9.2 (3.9)	10.1 (4.9)

HDL, high density lipoprotein; PHQ-9, Patient Health Questionnaire – nine items.

**Table 2.** Sample characteristics stratified by heart disease status

	Participants who did not develop heart disease *N* = 31 018	Participants who developed heart disease *N* = 2448
Age (Mean, SD)	52.7 (7.8)	56.4 (7.8)
Sex		
Female, % (*n*)	58.1 (18 015)	44.6 (1092)
Education		
Less than high school		
High school, % (*n*)	20.1 (6201)	26.0 (633)
College/graduate studies/ university, %(*n*)	79.9 (24 696)	74.0 (1801)
Ethnicity		
White, % (*n*)	88.6 (27 243)	90.2 (2183)
Smoking		
Current/occasional smoker, % (*n*)	16.5 (5106)	19.1 (465)
Former smoker, % (*n*)	39.0 (12 042)	44.1 (1076)
Never smoker, % (*n*)	44.5 (13 772)	36.9 (900)
Alcohol		
Daily alcohol consumption, % (*n*)	11.2 (3459)	12.7 (310)
Physical activity		
No moderate/vigorous activity, % (*n*)	19.3 (5101)	19.0 (380)
Diagnosed high blood pressure, % (*n*)	14.8 (4475)	28.6 (645)
Treated high blood pressure, % (*n*)	11.8 (3527)	24.4 (542)
Low cholesterol, % (*n*)	34.4 (9987)	43.5 (1025)
Diagnosed diabetes, % (*n*)	6.6 (2034)	13.0 (316)
Elevated depressive symptoms, % (*n*)	15.7 (4868)	19.2 (469)
Sleep disorder (previous 2 years), % (*n*)	3.0 (928)	4.4 (108)
Sleep duration		
Long sleep duration (⩾9 h), % (*n*)	9.6 (2974)	12.1 (295)
Short sleep duration (⩽6 h), % (*n*)	19.9 (6166)	21.9 (536)

Table 3 describes the HRs for heart disease across the depression–sleep disorders categories. Elevated depression symptoms and sleep disorders were independent predictors for heart disease when they were examined in separate analyses. When examined together with the NSND group as the reference group, the adjusted HR for heart disease was 2.60 (95% CI 1.83–3.69) for the SD group, 1.40 (95% CI 1.25–1.57) for the NSD group and 1.33 (95% CI 1.03–1.73) for the SND group. Test of additive interaction revealed an *S* of 2.17 (95% CI 1.01–4.64) in the adjusted model, suggesting a synergistic interaction between elevated depressive symptoms and sleep disorders under the additive model.

**Table 3.** Results of Cox regression analysis for associations between depressive symptom categories, diagnosed sleep disorders and heart disease

	Unadjusted model	Adjusted model
	HR (95% CI)	HR (95% CI)
Model 1: depression only		
No/low depressive symptoms	Reference	Reference
Elevated depressive symptoms	1.34 (1.21–1.48)	1.40 (1.25–1.56)
Model 2: diagnosed sleep disorder only		
No sleep disorder	Reference	Reference
Sleep disorder	1.65 (1.36–2.00)	1.52 (1.23–1.88)
Model 3: depression and diagnosed sleep disorder groups		
No/low depressive symptoms and no sleep disorder	Reference	Reference
No/low depressive symptoms and sleep disorder	1.52 (1.20–1.93)	1.33 (1.03–1.73)
Elevated depressive symptoms and no sleep disorder	1.31 (1.18–1.45)	1.40 (1.25–1.57)
Elevated depressive symptoms and sleep disorder	2.31 (1.67–3.20)	2.60 (1.83–3.69)

Adjusted model is adjusted for the following variables: age, sex, education, ethnicity, smoking status, alcohol consumption, physical activity, cholesterol, diagnoses diabetes and diagnosed high blood pressure.

The assumption of proportional hazards was examined. Comparison of observed *v.* expected survival curves within each group suggested that the assumption of proportional hazards was met.

The incidence rates were 17.0, 14.8 and 19.7 per 1000 persons per years for the short sleep, normal sleep and long sleep groups, respectively. HRs for heart disease across the depression and sleep duration categories are presented in Table 4. The group sizes for those without depression and with short, normal and long sleep durations were 5035, 20 481 and 2550, respectively. The group sizes for those with depression and with short, normal and long sleep durations were 1665, 2933 and 716, respectively. Both short sleep duration and long sleep duration were independent predictors for heart disease. Long sleep duration was associated with a higher risk for heart disease when elevated depressive symptoms were present: the adjusted HR increased from 1.16 (long sleep and no/low depressive symptoms) to 1.77 (long sleep and elevated depressive symptoms), with results of the contrast analysis suggesting that this difference was statistically significant (*χ*^2^ = 6.68, df = 1, *p* = 0.009). The presence of elevated depressive symptoms also increased the risk of heart disease for those with short sleep duration, although the difference was much smaller. The adjusted HR increased from 1.11 (short sleep duration without elevated depressive symptoms) to 1.35 (short sleep duration with the presence of elevated depressive symptoms), although the result of the contrast analysis was not statistically significant (*χ*^2^ = 2.91, df = 1, *p* = 0.088).

**Table 4.** Results of Cox regression analysis for associations between depressive symptom categories, sleep duration categories and heart disease

	Unadjusted model	Adjusted model
	HR (95% CI)	HR (95% CI)
Model 1: sleep duration categories only		
Normal sleep duration	Reference	Reference
Short sleep (⩽6 h)	1.15 (1.04–1.27)	1.11 (1.00–1.24)
Long sleep (⩾9 h)	1.39 (1.22–1.57)	1.21 (1.06–1.39)
Model 2: sleep duration categories and depression		
Normal sleep duration and no/low depressive symptoms	Reference	Reference
Normal sleep duration and elevated depressive symptoms	1.35 (1.17–1.54)	1.42 (1.23–1.65)
Short sleep (⩽6 h) and no/low depressive symptoms	1.14 (1.02–1.28)	1.11 (0.99–1.26)
Short sleep (⩽6 h) and elevated depressive symptoms	1.38 (1.16–1.64)	1.35 (1.12–1.63)
Long sleep (⩾9 h) and no/low depressive symptoms	1.35 (1.17–1.56)	1.16 (0.99–1.36)
Long sleep (⩾9 h) and elevated depressive symptoms	1.79 (1.42–2.25)	1.77 (1.37–2.28)

Adjusted model is adjusted for the following variables: age, sex, education, ethnicity, smoking status, alcohol consumption, physical activity, cholesterol, diagnoses diabetes and diagnosed high blood pressure.

The assumption of proportional hazards was examined. Comparison of observed *v.* expected survival curves within each group suggested that the assumption of proportional hazards was met.

Results of the sensitivity analyses are presented in Table 5. Overall, the sensitivity analyses demonstrated that (a) compared to the group with no depressive symptoms and no sleep disorders, all other sleep disorder and depressive symptom groups in the different sensitivity analyses had a higher risk of heart disease, with the highest risk found for the SD group; and (b) those with long sleep duration and elevated depressive symptoms had a higher risk for heart disease than those with normal sleep duration and no depressive symptoms. Information on central obesity, assessed by waist circumference, was only available in a subset of participants (mainly Phase A) and was therefore not included as a covariate in the main analyses, although a sensitivity analysis was conducted that included central obesity as an additional covariate in a subsample (*n* = 22 441; approximately 40% of the sample had central obesity). Adjusting for central obesity did not change the results substantively.

**Table 5.** Results of sensitivity analyses for the association between depressive symptom categories, diagnosed sleep disorders and heart disease

	Adjusted model
	HR (95% CI)
*Sensitivity analyses for sleep disorders*	
Analysis 1: diagnosed sleep disorders in the 5 years prior to baseline survey	
No/low depressive symptoms and sleep disorder	1.37 (1.13–1.65)
Elevated depressive symptoms and no sleep disorder	1.38 (1.22–1.55)
Elevated depressive symptoms and sleep disorder	2.42 (1.86–3.15)
Analysis 2: excluding sleep item from PHQ-9	
No/low depressive symptoms and sleep disorder	1.39 (1.09–1.77)
Elevated depressive symptoms and no sleep disorder	1.37 (1.20–1.57)
Elevated depressive symptoms and sleep disorder	2.67 (1.77–4.00)
Analysis 3: using PHQ-9 cut-off score of ⩾10	
No/low depressive symptoms and sleep disorder	1.43 (1.14–1.79)
Elevated depressive symptoms and no sleep disorder	1.36 (1.13–1.64)
Elevated depressive symptoms and sleep disorder	2.92 (1.72–4.95)
Analysis 4: competing-risk regression accounting for mortality	
No/low depressive symptoms and sleep disorder	1.33 (1.02–1.73)
Elevated depressive symptoms and no sleep disorder	1.38 (1.23–1.55)
Elevated depressive symptoms and sleep disorder	2.55 (1.79–3.65)
*Sensitivity analyses for sleep duration*	
Analysis 5: excluding sleep item from PHQ-9	
Normal sleep duration and no/low depressive symptoms	Reference
Normal sleep duration and elevated depressive symptoms	1.47 (1.24–1.73)
Short sleep (⩽6 h) and no/low depressive symptoms	1.15 (1.02–1.28)
Short sleep (⩽6 h) and elevated depressive symptoms	1.23 (0.96–1.56)
Long sleep (⩾9 h) and no/low depressive symptoms	1.19 (1.02–1.38)
Long sleep (⩾9 h) and elevated depressive symptoms	1.67 (1.25–2.22)
Analysis 6: using PHQ-9 cut-off score of ⩾10	
Normal sleep duration and no/low depressive symptoms	Reference
Normal sleep duration and elevated depressive symptoms	1.50 (1.17–1.94)
Short sleep (⩽6 h) and no/low depressive symptoms	1.12 (1.04–1.38)
Short sleep (⩽6 h) and elevated depressive symptoms	1.20 (0.88–1.63)
Long sleep (⩾9 h) and no/low depressive symptoms	1.19 (1.04–1.38)
Long sleep (⩾9 h) and elevated depressive symptoms	1.64 (1.11–2.42)
Analysis 7: competing-risk regression accounting for mortality	
Normal sleep duration and no/low depressive symptoms	Reference
Normal sleep duration and elevated depressive symptoms	1.41 (1.22–1.64)
Short sleep (⩽6 h) and no/low depressive symptoms	1.12 (0.99–1.26)
Short sleep (⩽6 h) and elevated depressive symptoms	1.34 (1.11–1.62)
Long sleep (⩾9 h) and no/low depressive symptoms	1.16 (0.99–1.36)
Long sleep (⩾9 h) and elevated depressive symptoms	1.71 (1.32–2.22)

All models are adjusted for the following variables: age, sex, education, ethnicity, smoking status, alcohol consumption, physical activity, cholesterol, diagnoses diabetes and diagnosed high blood pressure.

## Discussion

In this prospective community cohort study from Quebec, Canada, we evaluated the associations between depressive symptoms, sleep problems (i.e., diagnosed sleep disorders and short/long sleep durations) and the risk of incident heart disease in 33 455 participants without heart disease at baseline, by linking survey data with administrative data. We found that elevated depressive symptoms, diagnosed sleep disorders and short and long sleep duration were independent risk factors for heart disease. Diagnosed sleep disorders and elevated depressive symptoms also interacted to increase the risk of heart disease. Similarly, there was an interaction between long sleep duration and elevated depressive symptoms in relation to an increased risk of heart disease.

We found that individuals with both depressive symptoms and a diagnosed sleep disorder in the 2 years before baseline assessment had approximately 2.6 times increased risk of heart disease compared to those without either condition after accounting for sociodemographic, clinical and behavioural factors. The combined effect of the two conditions appeared to be greater than the sum of the individual effects, as suggested by the synergy index. A sensitivity analysis indicated that excluding the sleep item from the depressive symptom assessment yielded similar results as the main analysis, suggesting that the findings are not solely attributable to overlap in diagnostic criteria. The present results were generally consistent with prior research findings that have examined the independent associations between depressive symptoms (Van der Kooy *et al*., 2007) and sleep problems (Katz and McHorney, 1998; Bradley and Floras, 2009; Dikeos and Georgantopoulos, 2011) with the risk of heart disease. However, there is an overlap between depression and sleep problems, and the independent and combined effects of each condition have not previously been reported. Although a recent study demonstrated that, in a cohort of older adults from England, depression was associated with incident self-reported coronary heart disease independently from sleep problems, whereas sleep problems were not independently associated with coronary heart disease when accounting for depression (Poole and Jackowska, 2019), the potential synergistic association of sleep problems and depression was not examined.

This study also examined the associations between short, normal and long sleep durations with the risk of heart disease. The combination of depressive symptoms and long sleep duration was associated with the greatest risk of heart disease, with an approximate 79% increased risk of heart disease compared to those with no depressive symptoms and normal sleep duration. These findings were consistent with prior research findings. Long sleep duration has previously been shown to be associated with heart disease (Cappuccio *et al*., 2011; Gianfagna *et al*., 2016; Jike *et al*., 2018); our study extends this research by demonstrating that long sleep duration and elevated depressive symptoms are most strongly associated with heart disease when they co-occur. It is also possible that depressive symptoms mediate the association between long/short sleep duration and incidence of heart disease. For instance, sleep problems may increase the risk of depression which in turn increases the risk of heart disease. It is also possible that long/short sleep duration mediates the association between depressive symptoms and incidence of heart disease, such that depressive symptoms increase the risk of short/long sleep duration which in turn increases the risk of heart disease. Though it was not possible to examine the temporal nature of sleep duration and depressive symptoms in the association with heart disease in the present study, this question warrants future investigation.

Biological and behavioural pathways may explain the associations between depressive symptoms, sleep problems and the incidence of heart disease. Chronic inflammation may represent one pathway linking sleep and depressive symptoms to heart disease (McDade *et al*., 2006). For example, short sleep duration could result in increased inflammatory markers (Meier-Ewert *et al*., 2004), which are also associated with depression (Howren *et al*., 2009) and heart disease (Kaptoge *et al*., 2010). Alterations in hypothalamic–pituitary–adrenal activity might also be associated with psychological problems, sleep and heart disease (Antonijevic, 2008). Both disrupted sleep and depression are related to increased activity of the autonomic nervous system, which may represent another mechanism by which these factors confer risk for heart disease (Joynt *et al*., 2003). Long sleep paired with depression may represent an early manifestation of physical illness or a consequence of unrecognised chronic comorbidity. Long sleep duration has also been shown to be associated with fatigue and feelings of lethargy, which are common in depression and may be associated with the risk of heart disease (Grandner and Drummond, 2007). Sleep problems may also indicate a greater severity of depressive symptoms, with a greater risk of relapse and recurrence among those with sleep disturbances (Nutt *et al*., 2008), which may result in a more pronounced risk of heart diseases (e.g., Windle and Windle, 2013). Sleep apnoea treatments such as continuous positive airway pressure therapy have been shown to be associated with a lower risk of heart failure among those with sleep apnoea (Holt *et al*., 2018), and depression might interfere with adherence to sleep apnoea treatments (Law *et al*., 2014). Therefore, although we did not have information on treatments for sleep disorders, it is possible that poor adherence to such treatments might be a contributing mechanism for the synergistic association between sleep disorders and depression with the risk of heart disease.

Health behaviours such as smoking and physical inactivity are also potential mechanisms as they are linked with both sleep problems and depression (Mezick *et al*., 2011). The presence of both sleep problems and depressive symptoms might affect those health behaviours, which in turn increases the risk of heart disease. Finally, previous research has found that low socioeconomic status moderates the association between insomnia and heart diseases (Canivet *et al*., 2014) and depression and heart diseases (Lemogne *et al*., 2017) and might therefore also play a role in the synergistic interaction between depression and sleep problems with the risk of heart disease.

To our knowledge, this is the first study combining survey and administrative data to evaluate the joint effect of depressive symptoms and sleep problems on incident heart disease in individuals aged 40–69 years at baseline. The strengths of the present study include a large sample of middle-aged adults with up to 7 years of follow-up and two different measures of sleep problems. Several sensitivity analyses were further conducted to assess the robustness of the study findings.

There are also study limitations to acknowledge. Individuals with high depressive symptoms or diagnosed sleep problems in the past 2 years may have been treated at the time of the survey, and those with treated *v*. untreated (or not successfully treated) depressive symptoms or sleep disorders may have a different risk of heart disease. However, no information on treatment for depression or sleep disorders was available. In addition, no information on the duration of depressive symptoms or sleep problems was available, although the duration of depressive symptoms and sleep problems may impact heart disease risk.

The present study is also limited by the use of self-report to identify CARTaGENE participants with a lifetime history of heart disease at baseline, which was the main exclusion criterion for the present study. This selection criterion was based on self-report because the RAMQ data was limited to the past 10 years only and information was only available for those who lived in the province of Quebec during that period. Therefore, we did not have enough information to exclude participants based on RAMQ diagnoses and we could not compare the accuracy of self-reported diagnoses with objectively documented heart disease diagnoses. The assessment of comorbid conditions was also limited to self-report for the abovementioned reasons. On the other hand, the use of billing codes from the RAMQ for the assessment of heart disease could also be limited by the possibility of errors in coding.

The assessments of depressive symptoms and sleep duration were based on self-report as well. Detailed assessments of depressive symptomology and objective measures of sleep duration were not available. Recommendations have been made for the assessment of sleep duration to include objective and subjective measures (Van Den Berg *et al*., 2008). The lack of detail on each specific symptom of depression (i.e., weight loss *v.* weight gain and sleeping more *v.* less than usual) is another important limitation, given that depression is a heterogeneous construct with many possible symptom profiles (Fried and Nesse, 2015). Atypical depression is a subtype of depression, characterised by increased appetite or weight gain and longer sleep duration (American Psychiatric Association, 2013), which has previously been linked to cardiometabolic risk factors (Lasserre *et al*., 2017). It is possible that the combination of high depressive symptoms and long sleep duration in our study reflects the atypical subtype of depression, which could explain why long sleep duration paired with elevated depressive symptoms was associated with a higher HR for heart disease than short sleep duration with elevated depressive symptoms. However, there was insufficient information on depressive symptomology for the assessment of atypical depression in the present study. We also had insufficient power to examine specific subtypes of sleep diagnoses and heart disease diagnoses. In addition, the framing of the questions for the assessment of depressive symptoms limited to symptoms experienced in the past 2 weeks, and sleep duration was only assessed at one point in time. Therefore, the possible associations between changes in depressive symptoms and/or sleep problems with the risk of heart disease could not be examined in the present study. Our sample was also limited to mostly white participants, and therefore generalisability to more diverse ethnic groups cannot be established. Finally, as in all observational studies, there could be unmeasured confounding by unknown or unmeasured predictors.

Sleep problems are common comorbidities of depression, and an integrated approach that addresses both sleep problems and depression might be an important heart disease prevention strategy (Redline and Foody, 2011). Overall, the results of the present study highlight the interaction between depressive symptoms and sleep problems as a potentially important risk factor for heart disease and suggest that depression with sleep problems may be an important subtype of depression that is strongly associated with the risk of heart disease.

## Availability of Data and Materials

The data for this study are not publicly available, thus the authors do not have permission to share the data with outside parties that have not applied for access with the CARTaGENE research group. Requests for data access can be sent to the CARTaGENE research group (https://cartagene.qc.ca/).
